# A rapid point-of-care population-scale dipstick assay to identify and differentiate SARS-CoV-2 variants in COVID-19-positive patients

**DOI:** 10.3389/fmicb.2024.1459644

**Published:** 2024-10-21

**Authors:** Deepjyoti Paul, Jyoti Verma, Shakti Kumar, Daizee Talukdar, Pradipta Jana, Lekshmi Narendrakumar, Roshan Kumar, Subhash Tanwar, Mudita Gosain, Sonali Porey Karmakar, Madhu Pareek, Shailendra Mani, Susmita Chaudhuri, Pallavi Kshetrapal, Nitya Wadhwa, Shinjini Bhatnagar, Pramod Kumar Garg, Bhabatosh Das

**Affiliations:** ^1^Functional Genomics Laboratory, Centre for Microbial Research, Translational Health Science and Technology Institute, Faridabad, India; ^2^Maternal and Child Health, Translational Health Science and Technology Institute, Faridabad, India; ^3^Multidisciplinary Clinical and Translational Research, Translational Health Science and Technology Institute, Faridabad, India; ^4^Department of Gastroenterology and Human Nutrition, All India Institute of Medical Sciences, New Delhi, India

**Keywords:** SARS-CoV-2, COVID-19, next-generation sequencing, multiplex-PCR, dipstick assay, rapid detection

## Abstract

Delta and Omicron variants of Severe Acute Respiratory Syndrome Coronavirus 2 (SARS-CoV-2) are remarkably contagious, and have been recognized as variants of concern (VOC). The acquisition of spontaneous substitutions or insertion–deletion mutations (indels) in the spike protein-encoding gene substantially increases the binding affinity of the receptor binding domain (RBD)-hACE2 complex and upsurges the transmission of both variants. In this study, we analyzed thousands of genome sequences from 30 distinct SARS-CoV-2 variants, focusing on the unique nucleic acid signatures in the spike gene specific to the Delta and Omicron variants. Using these variant-specific sequences, we synthesized a range of oligonucleotides and optimized a multiplex PCR (mPCR) assay capable of accurately identifying and differentiating between the Delta and Omicron variants. Building on this mPCR assay, we developed a dipstick format by incorporating a tag linker sequence at the 5′ end of the forward primer and adding biotin to the 3′ end of the oligonucleotides, enhancing the assay’s usability and accessibility. Streptavidin-coated latex beads and the dipstick imprinted with a probe for the tag linker sequence in the test strips were used for the detection assay. Our dipstick-based assay, developed as a rapid point-of-care test for identifying and differentiating SARS-CoV-2 variants has the potential to be used in low-resource settings and scaled up to the population level.

## 1 Introduction

Following the first cases of SARS-CoV-2 in China during late 2019, a major outbreak of the coronavirus disease 2019 (COVID-19) was recorded worldwide, and the World Health Organization (WHO) declared this newly emerging infectious disease to be a pandemic. As of June 2023, more than 750 million cumulative cases have been registered in more than 220 nations and territories, resulting in over 6.9 million deaths (WHO COVID-19 dashboard, 2024). An enveloped virus with a single-stranded positive-sense RNA genome of about 30 kb is responsible for the illness, which is characterized by acute respiratory distress, fever, sore throat, muscle pain that can worsen into potentially fatal respiratory insufficiency while also having an impact on the neurological, heart, renal, and liver systems (Wang et al., 2020). In contrast to earlier coronaviruses like the Middle East Respiratory Syndrome Coronavirus (MERS-CoV) and the Severe Acute Respiratory Syndrome Coronavirus (SARS-CoV), the patient with the SARS-CoV-2 infection may experience a variety of clinical manifestations, from asymptomatic or mild infection to severe or critical illness like acute lung inflammation and pneumonia (V’kovski et al., 2021). The upper and lower respiratory tracts are primarily targeted by this pathogenic SARS-CoV-2, which also has a very high rate of human-to-human transmission, particularly in elderly and immunocompromised patients (Wolfel et al., 2020; Guan et al., 2020). Due to the symptoms’ resemblance to those of seasonal upper respiratory tract infections, accurate diagnostic techniques are required to identify viral nucleic acids, viral antigens, or serological assays that confirm SARS-CoV-2 infection.

The SARS-CoV-2 has tremendous competency to evolve rapidly by changing its genome sequences through accumulating substitutions or insertion–deletion mutations (indels) (Harvey et al., 2021). There have been multiple waves in the ongoing epidemic driven by emerging novel variants of SARS-CoV-2 variants. The genetic alterations brought on by various sorts of mutations, particularly in the spike protein, have resulted in the emergence of various lineages of the virus. The novel SARS-CoV-2 lineages have several fitness benefits, including increased virulence, infectivity, transmission, pathogenicity, immune evasion, and other fitness advantages, which could put a significant burden on public health systems. As a result, there is an unmet need for SARS-CoV-2 variants surveillance. The severity of COVID-19, the effectiveness of virus transmission, and the symptoms of sickness are all highly varied and strongly related to the different variants of SARS-CoV-2 (Tao et al., 2021). Additionally, there are significant variations in the SARS-CoV-2 incubation time and sustainability in the host. In order to impede the virus’s spread and stop the pandemic, community-level testing is crucial for identifying those who are infected and carrying the variant of concern (VOC) at an early stage. Viral transmission should always be stopped through contact tracing, clinical evaluation, and virus detection. To reduce the spread of SARS-CoV-2, a number of strategies including face masks, hand cleanliness, contact isolation, and social seclusion have already been implemented (Kevadiya et al., 2021).

**Figure 1:**
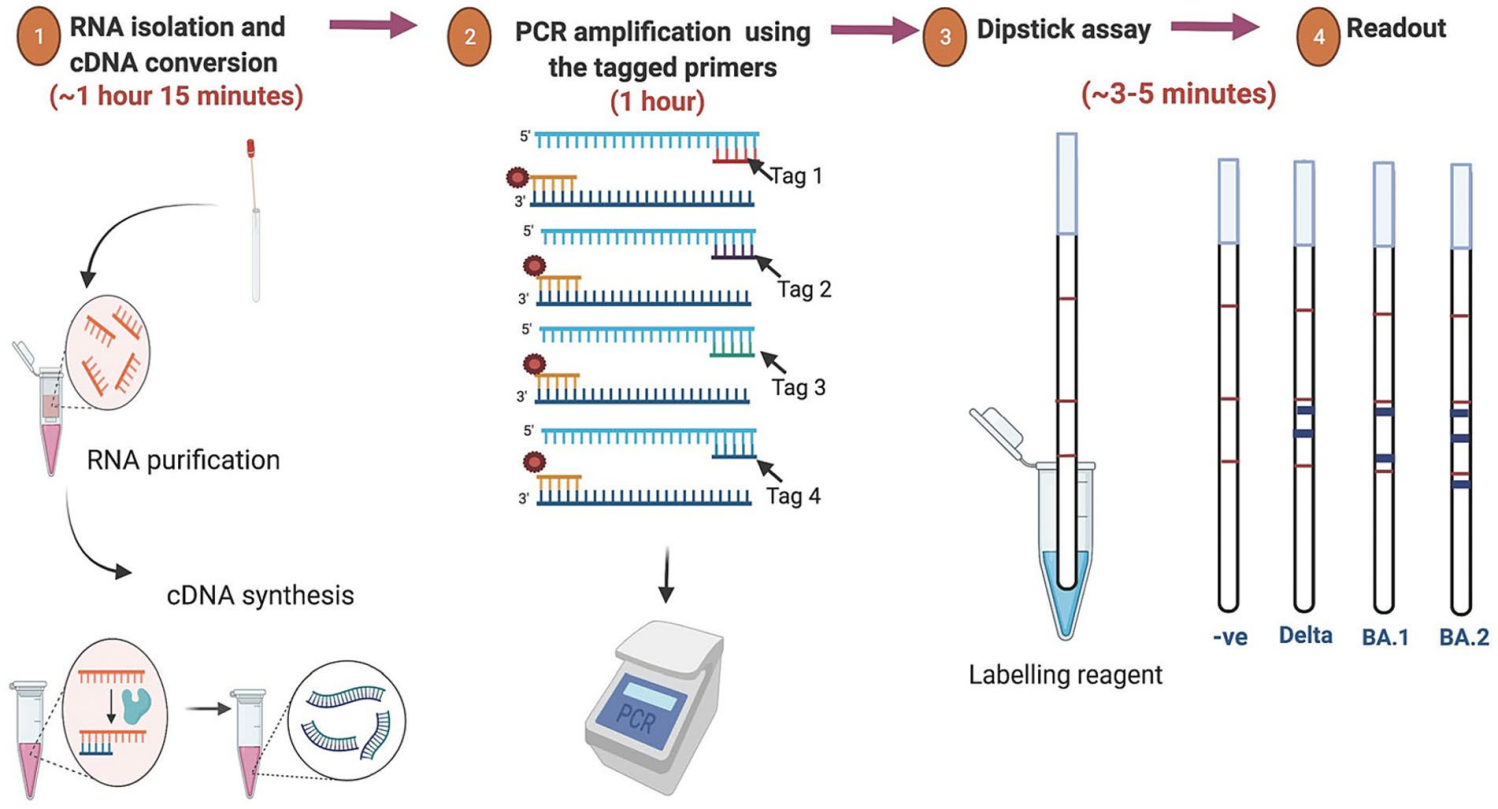
Overview of the assay for rapid detection of SARS-CoV-2 variants. The isolated RNA from the oro-nasopharyngeal swabs was used for cDNA synthesis followed by PCR amplification using the tagged variant-specific primer. For the dipstick assay the amplicon was mixed with a developing solution and avidin-coated blue beads. The results were interpreted based on the line location of the signal that appears after immersion of the dipstick.

Currently, the most common detection method for SARS-CoV-2 is the real-time reverse transcription polymerase chain reaction (RT-PCR) assay which utilizes the total RNA collected from the nasopharyngeal and/or oropharyngeal swabs samples of the suspected individuals (Guglielmi, 2021; Corman et al., 2020). However, the RT-PCR-based detection method has several limitations including the requirement of expensive thermal cyclers and reagents as well as its incapability to predict the virus variants. Moreover, the overall process from RNA isolation to result interpretation takes about 3–6 h depending on specific protocols used and laboratory efficiency. Furthermore, monitoring of SARS-CoV-2 lineages is often based on the sequencing of the entire viral genome or a subset of relevant regions (Meredith et al., 2020; Ko et al., 2022). This method calls for costly resources, technological know-how, and more time to process the data, leading to delayed result. The limited availability of specialized equipments and expertise further constrains genomic surveillance of variants in a densely populated nation like India. One of the primary factors contributing to the rapid spread of the virus is the absence of accessible and affordable diagnostic tools at the community level. For a rapid and wide-scale test at the community level, the test should be rapid, accurate, scalable, cost-effective, user-friendly and easy to interpret. One promising strategy for the rapid detection and screening of SARS-CoV-2 variants at the community level is identifying different and frequent minor indel mutations in the spike protein-encoding gene. Leveraging these mutations, we developed a rapid point-of-care test that can not only identify the presence of the SARS-CoV-2 virus in patients but also determine its variant. This test is particularly suited for low-resource settings, offering a practical alternative to RT-PCR. Remarkably, results can be interpreted in just 3–5 min following the amplification reaction, all without the need for sophisticated instruments. An overview of the SARS-CoV-2 dipstick assay is graphically represented in Figure 1.

## 2 Materials and methods

### 2.1 Sample collection and total RNA extraction

The total RNA was isolated from the oronasopharyngeal swab samples of SARS-CoV-2 positive patients using the QIAamp Viral RNA Mini Kit (Qiagen Cat. No.: 52906) to purify viral RNA for subsequent cDNA preparation and qRT-PCR. The sample was first lysed under highly denaturing conditions to inactivate RNases to ensure the isolation of intact viral RNA. Briefly, 140 μL of sample volume was lysed using 560 μL lysis buffer. Viral RNA was adsorbed onto the QIAamp silica membrane during 2 brief centrifugation steps. The RNA binds to the membrane, and contaminants were efficiently washed away in two steps using 2 different wash buffers AW1 and AW2. High-quality RNA was eluted in nuclease-free water, ready for direct use or safe storage at −80°C. All the steps were performed strictly according to the manufacturer’s protocol.

### 2.2 Detection of SARS-CoV-2 by RT-PCR assay from respiratory specimen

The COVIDsure Multiplex real-time RT-PCR Kit (Labsystems diagnostics, Cat No.:30201282) is a qualitative RT-PCR assay based on Taqman chemistry intended to be used for *in vitro* detection of SARS-CoV-2 (COVID-19) virus RNA in total RNA isolated from respiratory specimens (like nasopharyngeal swab, oropharyngeal swab) of suspected COVID-19 patients. The labeled primer-probe mix is specific to the SARS-CoV-2 genome (FAM labeled primer-probe set specific to Orf1ab and HEX labeled primer-probe set specific to E gene region). A signal in the FAM and/or HEX channel suggests presence of the SARS-CoV-2 virus in the sample. ROX labeled primer-probe set specific to RPP30 human gene was used as an internal control for RNA isolation, possible RT-PCR inhibition and to confirm the integrity of reagents of the kit. The assay was performed as per the manufacturer’s guidelines.

### 2.3 SARS-CoV-2 cultivation and viral RNA extraction

The virus culture experiments were conducted at the BSL-3 facility as approved by the Biosafety Committee of the research institute. Vero C1008 (Vero E6) cells (ATCC CRL-1586) were cultured in DMEM (Dulbecco’s Modified Eagle Medium) supplemented with 10% fetal bovine serum (FBS), MEM non-essential amino acids, 2 mM L-glutamine, 100 U ml^−1^ penicillin, 0.1 mg ml^−1^ streptomycin, 12.5 U ml^−1^ nystatin (Biological Industries) (Finkel et al., 2021). Briefly, the Vero E6 monolayers were washed once with DMEM without FBS and infected with SARS-CoV-2 virus, at a multiplicity of infection (MOI) of 0.2. After 1 h of infection, cells were cultured in DMEM medium supplemented with 2% FBS, MEM non-essential amino acids, L-glutamine and penicillin–streptomycin–nystatin at 37°C with 5% CO_2_. After four passages, titration was done on Vero E6 cells, followed by isolation of viral RNA from the infected cells which was then sequenced (details below).

### 2.4 Sequencing of SARS-CoV-2 genome

The pure virus isolates were harvested and RNA isolation was done using the QIAamp Viral RNA Mini Kit (Qiagen Cat. No.: 52906) and converted to cDNA. The viral genome from pure viral cDNA and clinical viral cDNA (Ct value<28) were sequenced accordingly as mentioned:

#### 2.4.1 Library construction

The isolated RNA was quantified using RNA HS Kit (Qubit, Cat No.: Q32855). 10–100 ng of purified total RNA was used as a starting material for the library construction. Briefly, the RNA was first denatured at 65°C for 5 min followed by priming of the random hexamers, which was followed by synthesis of the first strand of cDNA using RVT (reverse transcriptase). The RNA template was then removed followed by further synthesis of a replacement strand to generate blunt-ended, double stranded cDNA fragments at 16°C for 1 h. The synthesized double-stranded cDNA was cleaned up and tagmented using bead-linked transposomes at a temperature of 55°C for 5 min. The tagmentation process fragments the cDNA and adds index adapters to it. Illumina DNA/RNA UD indexes were used to index libraries. The tagmented DNA was further amplified by a specific PCR cycle, followed by clean-up using Agencourt Ampure XP beads (Make: Beckman Coulter, Cat No: A63881) and 80% ethanol. All the above methods were performed using Illumina® RNA Prep with Enrichment, (L) Tagmentation (Cat.no-20040537), IDT® for Illumina® DNA/RNA UD Indexes Set A, Tagmentation, Cat.no. – 20027213 (Illumina, California, US).

#### 2.4.2 Library enrichment and next-generation sequencing

The prepared libraries were then quantified using Qubit dsDNA BR assay kit and each library was normalized to 80 ng/μl. 3 plex enrichment was followed, based on their Ct values. Next, the probes were added to the 3 plex libraries to target regions of interest using the Respiratory Virus Oligo Panel (RVOP) enrichment. This enables the probes to get hybridized by incubating the reaction mixture overnight at 58°C (Respiratory Virus Oligos Panel V2, Cat.no – 20044311), (Illumina, California, US). Streptavidin magnetic beads were used to capture the hybridized probes ligated to the library fragments of interest. To remove any nonspecific binding, the beads were washed in a heated environment. Finally, the enriched library was then eluted from the beads. The amplification of the enriched library was done via a 14-cycle PCR program and finally the cleanup was performed using Agencourt Ampure XP magnetic beads. Each of the prepared libraries was checked firstly by Qubit quantification using Qubit dsDNA HS Kit. For the average library size, 1 μL enriched library was analyzed using the High sensitivity DS DNA Reagents (Make: Agilent, Cat No.: 5067–4,626) and run in Agilent 2,100 Bioanalyzer. All the 3-plexed libraries were normalized to 4 nM and pooled together, denatured and diluted as per the manufacturer’s protocol. The final 10pM denatured pooled library was loaded in the sample well of the Miseq reagent Kit V3 (150 cycles PE, Cat No.: MS-102-3001) (Illumina, California, US).

#### 2.4.3 Assembly and annotation of the SARS-CoV-2 genome

All SARS-CoV-2 genome was assembled based on reference-based assembling in which the Wuhan genome (GenBank ID: NC_045512) was used as a reference sequence. Three steps were followed during assembling the SARS-CoV-2 genome. (i) cleaning and merging the reads: All reads are quality checked by (Q_phred_ ≥ 30); checking and removal of the adapter and merging paired end reads to get longer raw reads by PEAR program.^1^ (ii) the reference-based assembling was performed by using the following tools *viz.* Hisat2,^2^ Samtools,^3^ bamToFastq,^4^ bcftools^5^ and seqtk.^6^ (iii) Annotation of the assembled SARS-CoV-2 genome by Prokka program^7^ based on the Wuhan genome (GenBank ID: NC_045512). All procedures and commands are available on the github link.^8^

### 2.5 Optimization of mPCR coupled dipstick assay conditions

The designed sets of primers specific for the SARS-CoV-2 and its variants were utilized to set up multiplex PCR in two distinct PCR sets (Set A & Set B). The two sets of PCR would differentiate between the Delta and Omicron (BA.1 & BA.2) variants. RdRp primers (RdRp-F& -R), BA.1 specific primer (BD3F &-R), and Delta specific primers (BD4-F & -R) were included in Set A of the mPCR mixture, along with other PCR reagents. Set B of the mPCR mixture included RdRp primers (RdRp- F& -R), Delta specific primers (BD4-F & -R), and BA.2 specific primers (BD6-F & -R) along with the PCR reagents. The PCR conditions were optimized as follows: initial denaturation of 95°C for 2 min followed by 28 cycles of denaturation at 95°C for 30 s, annealing at 55°C for 25 s and extension at 72°C for 45 s. After performing the final extension at 72°C for 2 min, the amplicon was collected and put through a dipstick assay.

### 2.6 Optimization of dipstick assay conditions

C-PAS (4) test strips (TBA Co., Ltd) for nucleic acid chromatography help in easy visualization of the amplified nucleic acid. Tagged and biotinylated primers were used for the amplification of the target genes. Amplicons were then mixed with the streptavidin-coated blue-colored latex beads and the developing solution. The immersion of the dipstick in the resulting solution generates a colored line (blue), which is specific to each tag and enables the detection of target genes easily. The volume of the developing solution and the latex beads were optimized to prevent any false positive results.

### 2.7 Screening of SARS-CoV-2 using the PCR-coupled dipstick method

A total of 75 samples were screened for SARS-CoV-2 using the PCR-coupled dipstick method. Four different sets of primers targeting the RdRp (positive control) and distinct regions of the spike protein encoding gene of Delta and Omicron (BA.1 and BA.2) variants were used for the screening. The screening involved PCR set-up followed by dipstick assay.

## 3 Results

### 3.1 Analysis of SARS-CoV-2 variants genome and identification of Delta and omicron specific genomic signature

**Figure 2:**
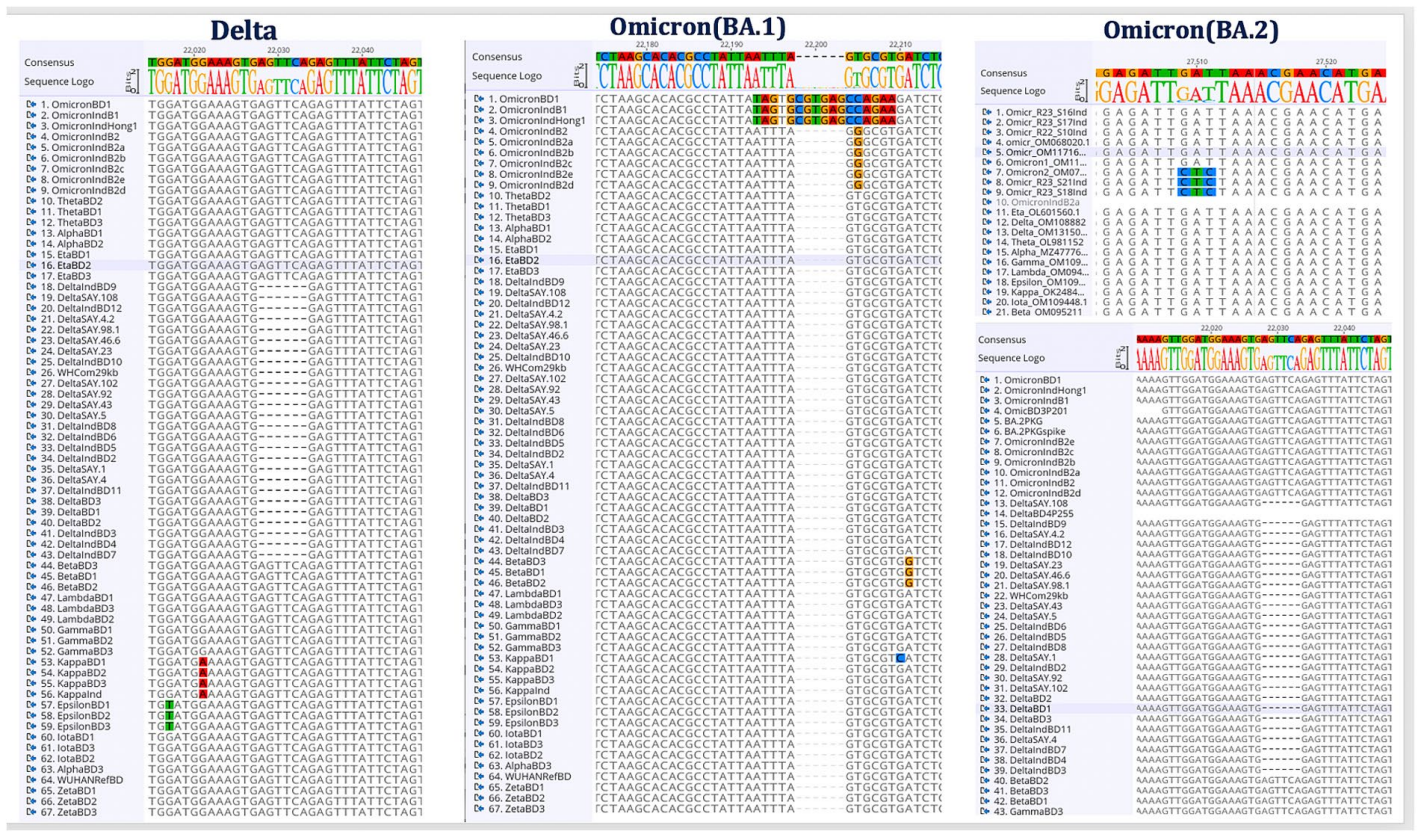
Identification of Delta and Omicron (BA.1 and BA.2) specific genomic signature sequences. Following the alignment of the genome sequences from several SARS-CoV-2 variants, the distinct genomic signatures for the Delta and Omicron (BA.1 and BA.2) variants were selected for the oligos design.

The genome sequences of different SARS-CoV-2 variants were obtained from GISAID (Global Initiative on Sharing Avian Influenza Data) (Khare et al., 2021) and the in-house Whole Genome Sequencing (WGS) facility of the institute. The sequences were analyzed using the Geneious software and the specific genomic signatures were identified for the prevalent Delta and Omicron variants. Detailed sequence analysis was performed and the characteristic Delta mutations and deletions were identified. The characteristic Omicron BA.1 ‘EPE’ insertion site was identified in the translated Spike protein. Furthermore, S-protein mutations of Omicron sublineage BA.2 were analyzed. The specific genomic signatures from the Delta, Omicron BA.1 and BA.2 spike protein-encoding genes are represented in Figure 2.

### 3.2 Oligos designing and in-silico genome amplification

Alignment of the spike protein-encoding gene sequences of the different SARS-CoV-2 variants was done and the specific genomic signatures of Delta, Omicron BA.1 and BA.2 variants were identified to design the specific oligos. The primers specific to the RdRp region were kept as a positive control. The designed oligos were checked for their specificity by in-silico genome amplification and were found to amplify only the specific target region. The nucleotide sequence and the specificity of the designed oligos is shown in Table 1.

**Table 1:** Oligos designed for the SARS-CoV-2 variants.

Primer ID	Sequence	Amplicon size (bp)	Specificity	Reference
RdRp-F RdRp-r	GTGARATGGTCATGTGTGGCGG CARATGTTAAASACACTATTAGCATA	100	All SARS-CoV-2 variants	Khare et al. (2021)
BD3-F BD3-R	GTTGGATGGAAAGTGAGTTCA GATCTTCTGGCTCACGCACTA	201	Omicron BA.1	This study
BD4-F BD4-R	GGTTCCATGCTATACATGT TGTTTTTGTGGTAATAAACAT	255	Delta & BA.2	This study
BD6-F BD6-R	AAAGTTGGATGGAAAGTGAG AACCCTGAGGGAGATCACG	210	Omicron BA.2	This study

### 3.3 Screening of SARS-CoV-2 positive samples and sequencing confirmation of the primer specificity

The designed sets of 4 primers were initially optimized for the amplification conditions in uniplex PCR using the respective control templates (pure virus cDNA and whole genome sequenced clinical samples). The specificity of the primers was determined by using the different viral cDNAs as the template. The primers successfully amplified the desired amplicon in optimized uniplex and multiplex PCR (mPCR) assays (Supplementary Figure S1). Optimal concentrations of each oligonucleotide primer set were optimized along with the cycle number for mPCR assays. The optimized conditions are mentioned in the Materials and methods section.

To establish the complete specificity of the oligos designed for this investigation, a total of 250 SARS-CoV-2 samples that were whole genome sequenced were screened using the oligos. The amplicons obtained was sequenced by Sanger sequencing and the sequence specificity was examined. The sequencing results matched perfectly with the WGS results confirming 100% specificity of the oligos designed in this study. The different samples screened and verified by these two methods have been mentioned in Table 2. The primer BD6, which is specific to the Omicron BA.2 variant was also tested for its binding to Omicron XBB variant. The oligos successfully amplified all the clinical samples identified as XBB 1.16 variant by Miseq Illumina sequencing.

**Table 2:** Samples submitted to WGS and further Sanger sequencing verification of the designed primer’s specificity.

Primer ID	Specificity	MiSeq (*n* = 250)	Sanger sequencing (*n* = 250)
RdRp- F & R	All SARS CoV-2 viruses	250	-
BD3- F & R	BA.1	23	23
BD4- F & R	Delta & BA.2	21 & 53	21 & 53
BD6- F & R	BA.2	206	206

### 3.4 Development of the probe and the dipstick assay

The primers designed for the specific SARS-CoV-2 variants: RdRp-F, BD3-F, BD4-F and BD6-F were labeled with the tag linker sequence (Tohoku Bio-array, Japan), complementary to the probe imprinted on the dipstick. The reverse primers: RdRp-R, BD3-R, BD4-R and BD6-R were labeled with biotin (Tohoku Bio-array) that binds to the streptavidin-coated blue latex beads (Tohoku Bio-array). The tag-linkers in the dipstick ensure that tagged PCR product specifically hybridizes to its complementary target sequences, enabling accurate detection and reducing false positives. Therefore, amplicons of the target gene will appear blue during the dipstick readout. For our PCR-coupled dipstick assay, PCR was performed with the labeled primers and 10 μL (5-fold diluted) of the amplicon was mixed with 10 μL of developing buffer containing 0 mM NaCl (Tohoku Bio-Array) and 1.2 μL of streptavidin-coated blue latex beads. Then, the dipstick was dipped into the reaction mixture for ~3–5 min. Since the dipstick is imprinted with the probe for the tag-linker sequence, the positive result was evident by the appearance of a blue line on the dipstick, which is easily visualized with the naked eye. The location of the blue line is distinct for the Delta, BA.1 and BA.2 variants.

### 3.5 Performance evaluation of RT-PCR coupled dipstick assay

**Figure 3:**
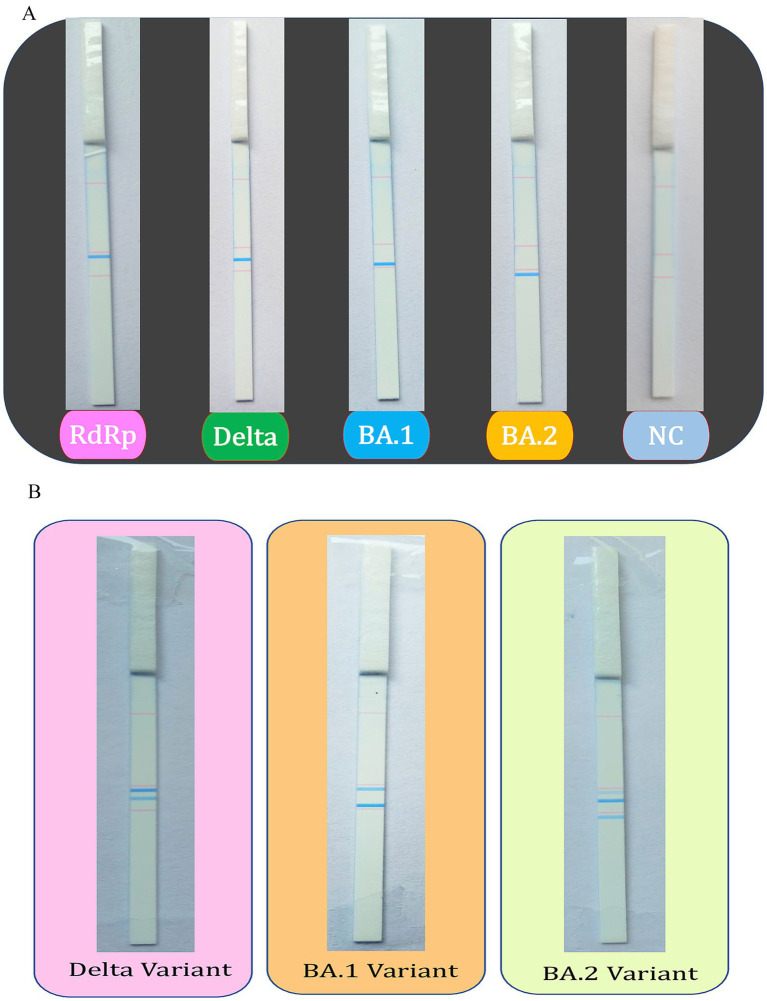
Dipstick assay results for the uniplex and multiplex PCR. The tagged oligos were used to screen the SARS-CoV-2 positive samples using **(A)** uniplex and **(B)** multiplex PCR, followed by the Dipstick assay. The interpretation of the results is aided by the line location as seen with the unaided eye.

The dipstick assay was used to examine 75 clinical specimens of SARS-CoV-2, which were whole genome sequenced and further verified by Sanger sequencing. The tagged oligos were utilized to screen the SARS-CoV-2 samples and their respective amplicons were used for the dipstick assay. The SARS-CoV-2 variants and positive samples were successfully identified by RT-PCR-coupled dipstick, hence, the dipstick readout was found to be totally specific. The presence of an RdRp-specific signal confirms the presence of SARS-CoV-2 and the other lines at distinct locations helps to infer the variant of SARS-CoV-2. The dipstick assay results of uniplex and multiplex PCR of SARS-CoV-2 samples have been shown in Figures 3A,B.

## 4 Discussion

More than four years have passed since the initial detection of SARS-CoV-2 and the World Health Organization’s declaration of the pandemic. Despite this time, COVID-19 remains uncontained, with the emergence and spread of new variants driven by specific mutations continuing to escalate. In response, we have designed our assay to target the unique mutations associated with each variant, specifically the presence of either the DEF156-157 mutation for Delta or the ins214EPE mutation for Omicron BA.1, enabling accurate detection and differentiation between these strains. The presence of this specific 214EPE insert has been successfully able to detect Omicron (B.1.1.529) in clinical samples (Phan et al., 2022). However, the upsurge in the number of cases and the emergence of BA.2 sub-lineage in many countries including India, significantly impelled to monitor its local transmission and this BA.2 differs from BA.1 by the absence of a specific signature for BA.1 ins214EPE and also additionally the presence of DLPP24-26.

Considering the spread and emergence of new SARS-CoV-2 variants, it is imperative to develop a rapid multi-variant detection assay of SARS-CoV-2. In the current work, a quick point-of-care test for the early detection of the SARS-CoV-2 virus and its variants was developed by understanding their specific genomic signatures. In contrast to the traditional real-time-based approach, which is complex and time-consuming, this standardized dipstick-based test is quick and straightforward. Additionally, while real-time PCR based approaches or other conventional approaches only determine the presence or absence of SARS-CoV-2 infection in a patient, our dipstick-based assay not just detects the virus, but also identifies the variant of SARS-CoV-2 simultaneously. Recently, a new recombinant Omicron XBB variant has emerged which is believed to have resulted from the combination of two lineages of BA.2 *viz.* BJ.1 and BM.1.1.1 (Tamura et al., 2023). This variant is known to exhibit high fitness and is likely to spread globally. The developed dipstick-based assay could also detect the newly emerged XBB variant. Hence, detection of this new lineage of SARS-CoV-2 using the Omicron BA.2 primers designed in the study could be used to assess the risk of SARS-CoV-2 infection effectively.

Recently, several rapid tests have been developed that guarantee the quick and resource-constrained identification of SARS-CoV-2 (Song et al., 2022). All of these tests, however, have some drawbacks, including the lack of specificity in antigen-based tests, inability to function in the presence of low viral loads, their lack of specificity, and the technical challenges with some of the tests. The tests that use nucleic acids are the most popular because they are extremely specific and can be used even when there is a low viral load (Udugama et al., 2020). However, these tests need greater time, expensive lab supplies, and apparatus. Though efforts have been made to simplify the nucleic acid-based tests by decreasing the time for RNA isolation, and cDNA conversion (Jorgensen et al., 2021; Lai et al., 2022), use of thermal incubators for isothermal amplification (Shanmugakani and Wu, 2022), use of CRISPR-Cas technology (Liu et al., 2022), potentiometric biosensors (Lee et al., 2022) and final readout using a colorimetric based assay with mobile application (Londono-Avendano et al., 2022) or the dipstick based results (Shanmugakani and Wu, 2022), none of the assays could differentiate the virus variants and were tested on large sample size. The rapid point-of-care test mentioned in this study outperforms the existing assays since it aids in variant identification (Delta, Omicron-BA.1 & BA.2) while also being accurate, robust, inexpensive, and quick. The test uses nucleic acid chromatography-based detection combined with tagged and labeled primers (Tohoku Bio-array, Japan). The results are clearly observable and interpretable to the naked eye and is apt for monitoring VOCs in environments with limited resources. Another major advantage of the developed assay is that it can be easily adaptable to include any new variants of SARS-CoV-2 that may potentially emerge in the future. Its rapid adaptability without the need for a complete re-design, ensures to keep the pace and the quick trace of the evolving virus, thereby enhancing public safety.

## 5 Conclusion

Effective management of the ongoing COVID-19 pandemic hinges on robust surveillance and testing. The tests utilized must be cost-effective, rapid, specific, and sensitive, especially in low-resource settings. This study presents a rapid point-of-care testing method for SARS-CoV-2 infection that can simultaneously identify various SARS-CoV-2 variants in patients, addressing the critical need for accessible and efficient testing solutions. Identifying circulating variants aids in tracking the spread of the virus and facilitates the early detection of potential outbreaks. Furthermore, accurately identifying variants helps assess vaccine effectiveness and helps development of new vaccine formulations. Additionally, recognizing the specific variants allows for the selection of the most effective therapeutic options and supports the reformulation of public health guidelines, ensuring preparedness for future outbreaks. The designed test is a nucleic-acid based dipstick assay, which is 100% specific and could be used as an alternative to qRT-PCR in low resource settings. The minimum requirement of the test includes the normal thermal cycler and its reagents along with the dipsticks. The results can be obtained in less time as compared to qRT-PCR without the need of any sophisticated instruments in just 3–5 min after the amplification reaction.

## Data Availability

The datasets presented in this study can be found in online repositories. The names of the repository/repositories and accession number(s) can be found at: https://ibdc.dbtindia.gov.in, INCARP000013, INCARP000014.

## Footnotes

^1^ https://cme.h-its.org/exelixis/web/software/pear/

^2^ http://daehwankimlab.github.io/hisat2/

^3^ http://www.htslib.org/

^4^ https://bedtools.readthedocs.io/en/latest/content/tools/bamtofastq.html

^5^ https://samtools.github.io/bcftools/bcftools.html

^6^ https://github.com/lh3/seqtk

^7^ https://github.com/tseemann/prokka

^8^ https://github.com/shakti83kumar/VGAP

## Supplementary material

SUPPLEMENTARY FIGURE S1 Electropherogram displaying PCR (uniplex and multiplex) amplification of Wuhan, Delta and Omicron variants. **(A)** An agarose gel image of a uniplex PCR utilizing primers that were specifically designed to target the RdRp gene and the spike protein-encoding gene of the Delta and Omicron (BA.1) variants. **(B)** Image of a multiplex PCR on an agarose gel using primers that were specifically designed to target the RdRp gene and the gene that codes for the spike protein in the Delta and Omicron (BA.1) variants.
