# Supplementary figures and images for: A rapid point-of-care population-scale dipstick assay to identify and differentiate SARS-CoV-2 variants in COVID-19-positive patients
Source: Front Microbiol. 2024 Oct 21;15:1459644. doi: 10.3389/fmicb.2024.1459644 (PMC11532176; doi:10.3389/fmicb.2024.1459644)

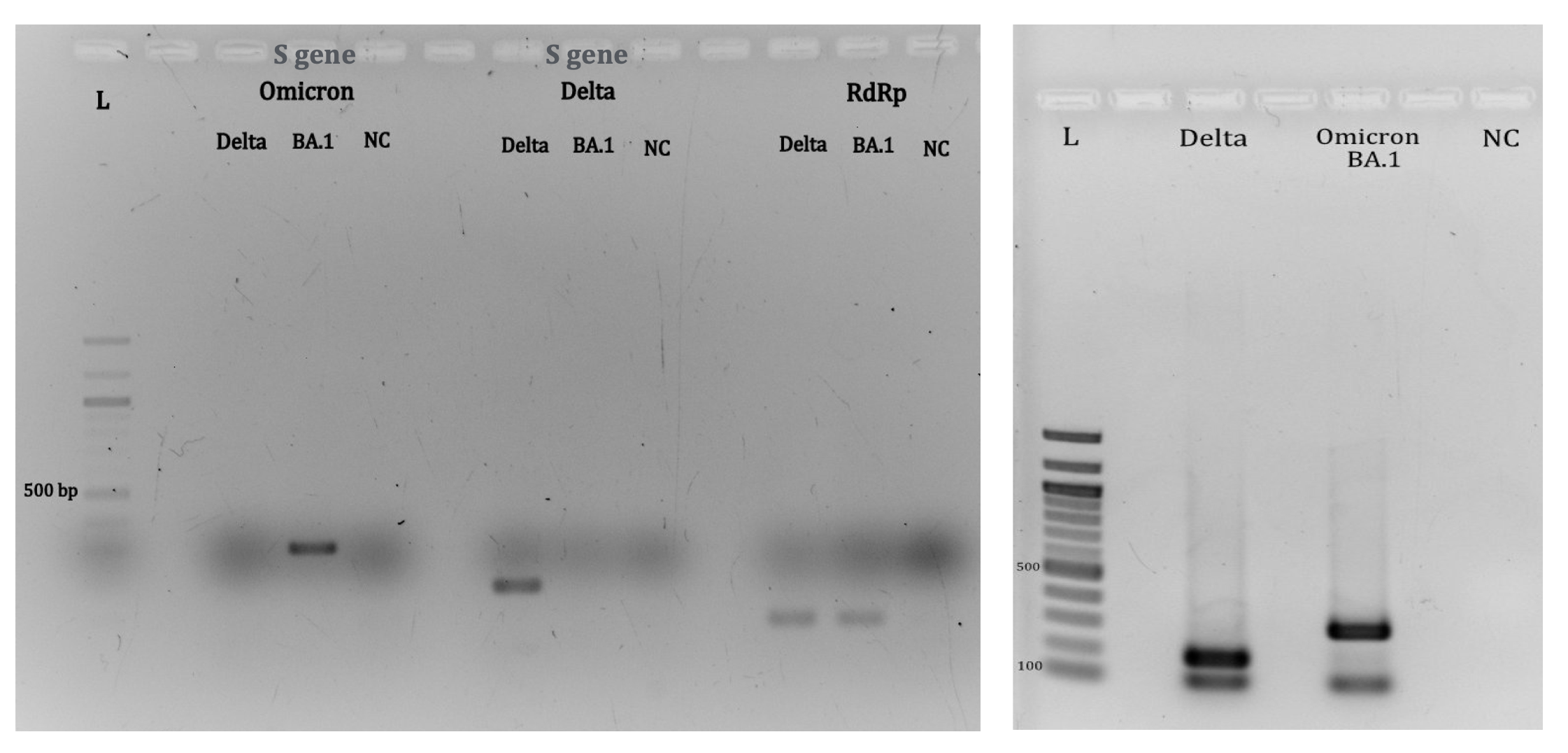

Supplement: SUPPLEMENTARY FIGURE S1 — Electropherogram displaying PCR (uniplex and multiplex) amplification of Wuhan, Delta and Omicron variants. (A) An agarose gel image of a uniplex PCR utilizing primers that were specifically designed to target the RdRp gene and the spike protein-encoding gene of the Delta and Omicron (BA.1) variants. (B) Image of a multiplex PCR on an agarose gel using primers that were specifically designed to target the RdRp gene and the gene that codes for the spike protein in the Delta and Omicron (BA.1) variants. [file Image_1.TIFF]
